# Infant gut microbiota and the hygiene hypothesis of allergic disease

**DOI:** 10.1186/1710-1492-8-S1-A12

**Published:** 2012-11-02

**Authors:** Meghan B Azad, Theodore Konya, Brenda Koster, Heather Maughan, David S Guttman, Catherine J Field, Radha S Chari, Malcolm R Sears, Allan B Becker, James A Scott, Anita L Kozyrskyj

**Affiliations:** 1 Pediatrics, University of Alberta, Edmonton, Alberta, Canada; 2 Dalla Lana School of Public Health, University of Toronto, Toronto, Ontario, Canada; 3 Cell & Systems Biology, University of Toronto, Toronto, Ontario, Canada; 4 Agriculture, Food & Nutritional Sciences, University of Alberta, Edmonton, Alberta, Canada; 5 Obstetrics & Gynecology, University of Alberta, Edmonton, Alberta, Canada; 6 Department of Medicine, McMaster University, Hamilton, Ontario, Canada; 7 Pediatrics & Child Health, University of Manitoba, Winnipeg, Manitoba, Canada; 8 Canadian Healthy Infant Longitudinal Development Study

## Background

Inverse associations between allergic disease and having pets or siblings are commonly attributed to the hygiene hypothesis. As an extension, one could posit that a less diverse gut microbiome in the infant, also linked with the development of allergic disease, would be a function of fewer microbes in the home environment. Piglet studies, however, indicate that greater microbe diversity in the environment actually leads to reduced diversity of the gut microbiota. In this study, we characterize the infant gut microbiota in relation to environmental factors traditionally associated with the hygiene hypothesis.

## Methods

The study comprised a small sub-sample of 24 infants from the Canadian Healthy Infant Longitudinal Development (CHILD) birth cohort. Birth mode was obtained from medical records, and mothers reported on pets, siblings, and breastfeeding. Fecal samples were collected at age 4 months, and microbiota composition was characterized by high-throughput 16S rRNA sequencing.

## Results

As reported by others, breastfed infants had lower microbiota richness and diversity compared to formula-fed infants. Microbiota richness and diversity were increased in infants living with pets, whereas these measures were decreased in infants with older siblings. Infants living with pets exhibited under-representation of Bifidobacteriaceae and over-representation of Peptostreptococcaceae; infants with older siblings exhibited under-representation of Peptostreptococcaceae.

## Conclusions

Two traditionally protective “hygiene hypothesis” factors have opposite effects on infant gut microbiota diversity, while apparently selecting for distinct combinations of intestinal microbes. This suggests that microbiota composition, rather than diversity, may be the more important driver of the “microflora hypothesis” of allergic disease.

